# On (*n*,*k*)-Simple Random Integer Lattices

**DOI:** 10.3390/e28060600

**Published:** 2026-05-27

**Authors:** Gengran Hu

**Affiliations:** 1 School of Cyberspace, Hangzhou Dianzi University, Hangzhou 310018, China; grhu@hdu.edu.cn; 2 Zhejiang Provincial Key Laboratory of Sensitive Data Security and Confidentiality Governance, Hangzhou Dianzi University, Hangzhou 310018, China

**Keywords:** (*n*,*k*)-simple, HNF, random integer lattice, rejection sampling method

## Abstract

Random integer lattices are fundamental to lattice-based cryptography and algorithmic number theory. A new random integer lattice model, free of any restrictions on the Hermite Normal Form (HNF), was introduced by in 2016. It was also observed that the probability of such a lattice being in a simple HNF form is approximately 44%. In this paper, the gap between general random integer lattices and those in a simple HNF is bridged by introducing the concept of the (n,k)-simple random integer lattice, where the first *k* diagonal entries of the HNF are fixed to 1. We derive the asymptotic counting formula for such lattices and compute their density among all integer lattices. Furthermore, a generation algorithm for the (n,k)-simple random integer lattice based on rejection sampling and inverse sampling methods are proposed, with the analysis showing that it achieves $O(n^{2})$ expected running time. This work provides a theoretical foundation and practical toolkit for constructing structured random lattices with controlled HNF forms.

## 1. Introduction

The study of random integer lattices is significant in computational number theory and cryptography, particularly in the analysis of lattice-based cryptographic primitives such as NTRU [1], LWE [2], and their variants [3,4]. A challenge in this area lies in understanding the distribution of lattice bases and their associated Hermite Normal Form (HNF), which offers a unique normal form for integer lattices [5]. HNF has been studied to some extent in algorithmic number theory. Early work by Kannan and Bachem [6] established polynomial-time algorithms for HNF computation, while subsequent research by Micciancio and Warinschi [7] explored its applications in cryptography. The probabilistic properties of HNF have attracted considerable attention, with studies on the distribution of diagonal entries and their statistical behavior [8].

The problem of generating random lattices has been studied from multiple perspectives. Goldstein and Mayer [9] studied the distribution of lattice determinants and related arithmetic functions, laying the foundation for probabilistic models of lattices. Concerning HNF of integer matrices, Maze [10] explored their natural density distribution. Then the enumeration of integer lattices with a bounded determinant was systematically studied by Hu et al. [11] and later refined in [12] on the generation algorithm, providing the theoretical basis for random lattice models. Especially in [11], the analysis revealed that a significant proportion—approximately 44% of such random lattices—already exhibit a simple HNF structure, where the diagonal entries are all 1 but the last one. However, this observation raises a natural question: what is the precise probability that an HNF has its first *k* diagonal entries equal to 1, and how can such structured lattices be generated efficiently?

When cryptographic applications are considered, the generation of random lattices with specific properties has become increasingly important. The NTRU cryptosystem [1] relies on lattices with special algebraic structures, while LWE-based schemes [2] often require random lattices with controlled parameters. Later works on efficient lattice trapdoors [13], Ring-LWE toolkit [14], FHE based on LWE and NTRU [15], and lattice-based obfuscation [16] have further emphasized the need for efficient and flexible lattice generation methods. Meanwhile, to assess the security of lattice-based cryptosystems, algorithms like LLL [17] and BKZ [18,19] are commonly employed. Since both cryptographic constructions and the corresponding lattice reduction algorithms operate on integer lattices, which are discrete subgroups of $Z^{n}$, the question of how to properly define and randomly generate such lattices is of great importance. Once a method for generating random integer lattices is constructed, it can be employed to supply random inputs to lattice algorithms, thereby allowing their average performance characteristics to be evaluated.

In this paper, we formalize the notion of (n,k)-simple HNF, defined as an n×n HNF whose first *k* diagonal entries are 1. This definition fills the gap between the general random integer lattice model and the special case of simple HNF considered in [11]. We study the asymptotic enumeration of such lattices and derive closed-form expressions for their density among all integer lattices. Our results generalize the earlier findings and provide a unified framework for analyzing HNF-restricted random lattices. For example, the well-known NTRU lattice, derived from the public key polynomial h(x) in NTRU cryptosystem, has the basis matrix of the form $B_{NTRU}=\left(\begin{array}{cc} I_{N} & H\\ 0 & qI_{N} \end{array}\right),$ where $I_{N}$ denotes the N×N identity matrix, *q* is the modulus, and *H* is an N×N circulant matrix generated by the degree-*N* polynomial h(x) with small integer coefficients. The security of NTRU cryptosystem lies in the hardness of finding the private key, which is some short vector in the lattice $L(B_{NTRU})$. To be more specific, we consider a concrete tiny instance of NTRU. Let N=4 and the modulus q=17; suppose that the circulant matrix *H* is given by

$$H=\left(\begin{array}{cccc} 1 & 1 & 0 & 1\\ 1 & 1 & 1 & 0\\ 0 & 1 & 1 & 1\\ 1 & 0 & 1 & 1 \end{array}\right),$$

where *H* corresponds to the ideal generated by the polynomial $1+x+x^{3}$ (matching the first row (1,1,0,1) of the matrix) in the ring $Z\left[x\right]/\left(x^{N}-1\right)$; then the corresponding NTRU lattice basis matrix $B_{NTRU}$ is an 8×8 matrix constructed as

$$B_{NTRU}=\left(\begin{array}{cccccccc} 1 & 0 & 0 & 0 & 1 & 1 & 0 & 1\\ 0 & 1 & 0 & 0 & 1 & 1 & 1 & 0\\ 0 & 0 & 1 & 0 & 0 & 1 & 1 & 1\\ 0 & 0 & 0 & 1 & 1 & 0 & 1 & 1\\ 0 & 0 & 0 & 0 & 17 & 0 & 0 & 0\\ 0 & 0 & 0 & 0 & 0 & 17 & 0 & 0\\ 0 & 0 & 0 & 0 & 0 & 0 & 17 & 0\\ 0 & 0 & 0 & 0 & 0 & 0 & 0 & 17 \end{array}\right).$$

Since $B_{NTRU}$ is already in upper triangular form with positive diagonal entries, and the upper triangular entries satisfy ${\left(B_{NTRU}\right)}_{ij}\in \left[0,{\left(B_{NTRU}\right)}_{jj}\right]$ for i<j, while the first k=N=4 diagonal entries are 1, this specific basis represents an exact (8,4)-simple HNF and the corresponding integer lattice. This framework can be used to rigorously evaluate the average-case performance of lattice reduction algorithms on this structured family by generating uniformly random instances within the controlled (n,k)-simple HNF space, rather than relying on completely unstructured random integer lattices.

The key advances of this work can be summarized in the following.

1. The concept of an (n,k)-simple random integer lattice is introduced and its asymptotic counting formula is established.
2. The density of such lattices among all integer lattices is derived, expressed in terms of the Riemann zeta function.
3. An efficient generation algorithm combining rejection sampling and continuous inverse sampling is proposed, with a rigorous analysis of its time complexity.

This paper proceeds as follows. Section 2 recalls the basic notions of lattices and HNF. Section 3 gives the asymptotic counting formula for (n,k)-simple random integer lattices. Section 4 computes the density of such lattices. Section 5 details a generation algorithm along with its complexity. Section 6 closes with concluding remarks.

## 2. Preliminaries

### Lattice and HNF

Let $B\in R^{m\times n}$ with m≤n and rank(B)=m, the lattice spanned by the basis *B* is

$$L\left(B\right)=\left\{\sum_{i=1}^{m}x_{i}b_{i}|x_{i}\in Z\right\},$$

with $b_{i}$ the *i*-th row of *B*. The lattice has rank *m*. When m=n, the lattice is called full-rank, and the lattice determinant is defined as det(L(B))=|det(B)|. In the rest of this paper, all lattices are assumed to be full-rank.

A unimodular matrix U∈GL(n,Z) satisfies $B=UB^{\prime }$ if and only if the two bases *B* and $B^{\prime }$ span the same lattice. Cryptographic schemes usually consider integer lattices, meaning their basis matrices have entries in Z rather than R. Hence, the space of all full-rank integer lattices is the quotient $GL\left(n,R\right)\cap Z^{n\times n}/GL\left(n,Z\right)$.

HNF is a special type of integer matrix used in number theory, whose definition is as follows.

**Definition 1**  (HNF)**.** *An n×n integer matrix is called an n×n HNF when it meets the following criteria:*

- *Upper triangular: $h_{ij}=0$ for all indices with i>j;*
- *Positive diagonal: $h_{ii}>0$ for each i;*
- *Modular reduction: For any off-diagonal entry above the diagonal, it requires that $h_{ij}\in \left[0,h_{jj}\right)$ for all i<j.*

Now we define a family of special form of HNF, called (n,k)-simple HNF, which is described below.

**Definition 2**  ((n,k)-simple HNF)**.** *An n×n integer matrix H is called an (n,k)-simple HNF if it meets the following criteria:*

- *H is an n×n HNF;*
- *The first k diagonal entries of H, or $h_{11},h_{22},\cdots ,h_{kk}$ are *1*.*

If a random HNF is chosen, most of its diagonal entries will be 1 [11]; therefore, it is necessary to consider the special form of HNF with several 1s on the diagonal.

## 3. The (*n*,*k*)-Simple Random Integer Lattice

It is a classical result that every full-rank integer lattice possesses a unique HNF as its basis [20], and by [11], the random integer lattice is defined by an HNF chosen uniformly from the set $H_{n}^{\leq }\left(M\right)$, where $M\in Z^{+}$ is large enough, and

$$H_{n}^{\leq }\left(M\right)≜\left\{H|H\;\mathrm{is}\;\mathrm{an}\;n\times n\;\mathrm{HNF}\;\mathrm{with}\;\mathrm{determinant}\leq M\right\}.$$

Here we generalize this definition to the set of (n,k)-simple form of the HNF, which is stated as

$$H_{n,k}^{\leq }\left(M\right)≜\left\{H|H\;\mathrm{is}\;\mathrm{an}\;\left(n,k\right)\text{-}\mathrm{simple}\;\mathrm{HNF}\;\mathrm{with}\;\mathrm{determinant}\leq M\right\}.$$

From [11], we already knew the asymptotic formula for $|H_{n}^{\leq }\left(M\right)|$, which is

(1) $$|H_{n}^{\leq }\left(M\right)|=\frac{\prod_{j=2}^{n}\zeta \left(j\right)}{n}M^{n}+O\left(M^{n-1}\log M\right),$$

now we intend to obtain the asymptotic formula for $H_{n,k}^{\leq }\left(M\right)$ on k=1,⋯,n−1. First, we need a lemma in [11] that counts the summation of certain power series, which is given below.

**Lemma 1**  (From [11], Lemma 1)**.** *For a given integer n≥4 and sufficiently large $M\in Z^{+}$, let ${\left(t_{i}\right)}_{i=1}^{n}$ be a non-decreasing sequence of non-negative integers satisfying $t_{n-3}\leq t_{n-2}<t_{n-1}\leq t_{n}$. If the multiple sum is defined as*

$$S\left(M,t_{1},\cdots ,t_{n}\right)≜\sum_{a_{i}\in Z^{+},\prod_{i=1}^{n}a_{i}\leq M}a_{1}^{t_{1}}\cdots a_{n}^{t_{n}},$$

*where ζ(s) is the Riemann zeta function, then the asymptotic expansion of $S(M,t_{1},\cdots ,t_{n})$ is given as*

$$S\left(M,t_{1},\cdots ,t_{n}\right)=\left\{\begin{array}{c} \frac{\prod_{j=1}^{n-1}\zeta \left(s_{n}+1-s_{j}\right)}{s_{n}+1}M^{s_{n}+1}+O\left(M^{s_{n}}\log M\right),\;\;\;\;\;\;t_{n-1}<t_{n}\\ \frac{\prod_{j=1}^{n-1}\zeta \left(s_{n}+1-s_{j}\right)}{s_{n}+1}M^{s_{n}+1}\log M+O\left(M^{s_{n}+1}\right),\;\;\;t_{n-1}=t_{n} \end{array}\right..$$

Using Lemma 1, we can obtain the counting result on $|H_{n,k}^{\leq }\left(M\right)|$, which is shown in Theorem 1.

**Theorem** **1.** *For integer n≥4 and sufficiently large $M\in Z^{+}$, the value of $|H_{n,k}^{\leq }\left(M\right)|$ can be estimated asymptotically by*

(2) $$|H_{n,k}^{\leq }\left(M\right)|=\frac{\prod_{j=2}^{n-k}\zeta \left(j\right)}{n}M^{n}+O\left(M^{n-1}\log M\right).$$

**Proof.**  By definition, any $H\in H_{n,k}^{\leq }\left(M\right)$ should satisfy that *H* is (n,k)-simple and det(H) is bounded by *M*. Since the (n,k)-simple property means that the first *k* diagonal entries of *H* are exactly 1, we know that $|H_{n,k}^{\leq }\left(M\right)|$ is determined exclusively by $a_{k+1},\cdots ,a_{n}$, which is represented by the following formula as

$$\sum_{a_{i}\in Z^{+},\prod_{i=k+1}^{n}a_{i}\leq M}1^{0}\cdots 1^{k-1}a_{k+1}^{k}a_{k+2}^{k+1}\cdots a_{n}^{n-1}.$$

By Lemma 1, we obtain

$$\begin{array}{cc} |H_{n,k}^{\leq }\left(M\right)| & =\sum_{a_{i}\in Z^{+},\prod_{i=k+1}^{n}a_{i}\leq M}a_{k+1}^{k}a_{k+2}^{k+1}\cdots a_{n}^{n-1}\\ \; & =S(M,k,k+1,\cdots ,n-1)\\ \; & =\frac{\prod_{j=1}^{n-k-1}\zeta \left(n-\left(k-1+j\right)\right)}{n}M^{n}+O\left(M^{n-1}\log M\right)\\ \; & =\frac{\prod_{j=2}^{n-k}\zeta \left(j\right)}{n}M^{n}+O\left(M^{n-1}\log M\right). \end{array}$$

The proof is hereby completed.    □

Now we give the definition of (n,k)-simple random integer lattice as follows.

**Definition 3**  ((n,k)-simple random integer lattice)**.** *For integer n≥4, and the determinant upper bound $M\in Z^{+}$, if H is selected uniformly from $H_{n,k}^{\leq }\left(M\right)$, then L(H) is called an (n,k)-simple random integer lattice.*

**Remark** **1.** *It should be pointed out that when k=0, the (n,0)-simple random integer lattice is exactly the random integer lattice defined in [11].*

## 4. The Density of (*n*,*k*)-Simple Integer Lattice

After defining the (n,k)-simple random integer lattice, the following theorem gives the density of the (n,k)-simple integer lattice in all of the integer lattices.

**Theorem** **2.** *For integer n∈[4,+∞), and a large enough $M\in Z^{+}$, when M→+∞, let H be uniformly chosen from $H_{n}^{\leq }\left(M\right)$; then the probability of H being (n,k)-simple is $1/\prod_{j=n-k+1}^{n}\zeta \left(j\right)$ for k=1,2,⋯,n−1.*

**Proof.**  Combing the results from Equations (1) and (2), we obtain the following

$$\begin{array}{cc} \; & Prob(H\;\mathrm{is}\;\left(n,k\right)\text{-}\mathrm{simple})\\ = & \frac{|H_{n,k}^{\leq }\left(M\right)|}{|H_{n}^{\leq }\left(M\right)|}\\ = & \frac{\frac{\prod_{j=2}^{n-k}\zeta \left(j\right)}{n}M^{n}+O\left(M^{n-1}\log M\right)}{\frac{\prod_{j=2}^{n}\zeta \left(j\right)}{n}M^{n}+O\left(M^{n-1}\log M\right)}\\ = & \frac{1/\prod_{j=n-k+1}^{n}\zeta \left(j\right)+O\left(\log M/M\right)}{1+O(\log M/M)}\\ = & 1/\prod_{j=n-k+1}^{n}\zeta \left(j\right)+O\left(\log M/M\right), \end{array}$$

implying that when *M* tends to infinity, the probability of *H* being (n,k)-simple is $1/\prod_{j=n-k+1}^{n}\zeta \left(j\right)$, which completes the proof.    □

More clearly, the approximate numerical result on the density of (n,k)-simple integer lattices from Theorem 2 is shown in Table 1, where *n* is set to $2^{s}$ for s∈[3,8], and *k* is set to $1,\frac{n}{4},\frac{n}{2},\frac{3n}{4}$ and n−1.

Based on the data presented in Table 1, for a fixed lattice rank *n*, the density decreases monotonically as *k* increases, since imposing more diagonal entries equal to 1 introduces stricter structural constraints, thus reducing the number of admissible lattices. In more detail, when *k* is relatively small compared to *n*, the density rapidly approaches 1 as *n* increases. For instance, at k=n/2, the density rises from approximately 0.96145 for n=8 to almost 1.00000 for n=64,128,256. This indicates that a random integer lattice with high rank almost surely satisfies the condition that the first *k* diagonal entries of its HNF are equal to 1. Moreover, when k=n−1, the density stabilizes at approximately 0.43576 and exhibits nearly no dependence on *n*. This limiting value coincides with the theoretical constant $\prod_{j=2}^{+\infty }\zeta \left(j\right)$.

## 5. The Generation Algorithm of (*n*,*k*)-Simple Random Integer Lattice

Since in [12], the authors proposed an algorithm aimed at generating a random integer lattice, in this part, we generalize this algorithm to the (n,k)-simple random integer lattice for k∈[1,n−1].

### 5.1. Rejection Sampling and Inverse Sampling

To illustrate this algorithm clearly, we first need two fundamental techniques from probability theory: rejection sampling and continuous inverse sampling. When dealing with a distribution *D* that is defined on a set *A*, these methods can produce a random variable that adheres precisely to *D*. The following two theorems outline these two standard sampling methods respectively. For rigorous proofs and detailed theoretical foundations, we refer the reader to [21] and [22], respectively.

**Theorem 3**  (Rejection Sampling, from [21])**.** *Suppose D is a distribution defined on a set A with its density function f(x), $D^{\prime }$ is another distribution with proposal density function g(x). Let C be a constant s.t. f(x)≤Cg(x) for all x. Then iteratively do the following: Sample $x∼D^{\prime }$ and u∼U[0,1]; if $u>\frac{f(x)}{Cg(x)}$, reject and repeatedly sample x and u; otherwise accept and output x. Finally, the output x then follows the target distribution D.*

**Theorem 4**  (Inverse Sampling, from [22])**.** *Let D be a distribution defined over a set A with a cumulative distribution function F. Sample y∼U[0,1] and compute x satisfying F(x)=y. Then the random variable x follows the target distribution D.*

### 5.2. Generating the (n,k)-Simple Random Integer Lattice

As discussed in Section 2 and Section 3, an integer lattice has a unique HNF. Since an (n,k)-simple random integer lattice has the first *k* diagonal entries $a_{1}=\cdots =a_{k}=1$, its leading k×k principal submatrix has already been determined to be $I_{k}$. The remaining generation process consists of three stages. First, the diagonal entries $a_{k+1},a_{k+2},\cdots ,a_{n-1}$ are generated. Second, the final diagonal entry $a_{nn}$ is generated. Third, all off-diagonal entries are chosen. The resulting matrix *H* is then output as the basis matrix for the (n,k)-simple random integer lattice.

#### 5.2.1. Generating the Diagonal Entries $h_{k+1,k+1},\cdots ,h_{n-1,n-1}$

In this part, to generate the diagonal entries $h_{k+1,k+1},\cdots ,h_{n-1,n-1}$, the distribution of these variables should be obtained. The joint distribution of $a_{k+1},\cdots ,a_{n-1}$ is described below.

**Theorem** **5.** *Given the lattice rank n≥4 and the upper bound of the lattice determinant $M\in Z^{+}$, if $H=(h_{ij})$ is an (n,k)-simple random integer lattice with k∈[1,n−1], then for $b_{1},\cdots ,b_{n-k-1}\in Z^{+}$, when M→+∞, for i∈[1,n−k−1], it can be obtained that*

$$\mathrm{Prob}\left(h_{k+i,k+i}=b_{i}\;\mathrm{for}\;\mathrm{all}\;i\right)=\frac{\prod_{i=1}^{n-k-1}b_{i}^{-(n+1-i)}}{\prod_{j=2}^{n-k}\zeta \left(j\right)}.$$

**Proof.**  Since the diagonal entries $a_{k+1},\cdots ,a_{n-1}$ have been set to $b_{1},\cdots ,b_{n-k-1}$ respectively, the only free variable to do summation is the last diagonal entry $a_{nn}$. From Lemma 1, we know that

$$\begin{array}{cc} \; & Prob(h_{k+1,k+1}=b_{1},\cdots ,h_{n-1,n-1}=b_{n-k-1})\\ = & \lim_{M\to +\infty }\frac{\left(\sum_{a_{1}\leq M/\left(b_{1}\cdots b_{n-k-1}\right)}a_{1}^{n-1}\right)\prod_{i=1}^{n-k-1}b_{i}^{k+i-1}}{|H_{n,k}^{\leq }\left(M\right)|}\\ = & \lim_{M\to +\infty }\frac{\left(\frac{1}{n}{\left(\frac{M}{b_{1}\cdots b_{n-k-1}}\right)}^{n}+O\left({\left(\frac{M}{b_{1}\cdots b_{n-k-1}}\right)}^{n-1}\log\frac{M}{b_{1}\cdots b_{n-k-1}}\right)\right)\prod_{i=1}^{n-k-1}b_{i}^{k+i-1}}{\frac{\prod_{j=2}^{n-k}\zeta \left(j\right)}{n}M^{n}+O\left(M^{n-1}\log M\right)}\\ = & \frac{\prod_{i=1}^{n-k-1}b_{i}^{-(n+1-k-i)}}{\prod_{j=2}^{n-k}\zeta \left(j\right)}, \end{array}$$

which completes the proof.    □

Moreover, the distribution of each value of $a_{k+1},\cdots ,a_{n-1}$ is stated below.

**Theorem** **6.** *Consider an integer lattice of rank n≥4 with a prescribed upper bound $M\in Z^{+}$ on its determinant. If $H=(h_{ij})$ is an (n,k)-simple random integer lattice with k∈[1,n−1], then for $b_{1},\cdots ,b_{n-k-1}\in Z^{+}$, when M→+∞, for each i∈[1,n−k−1], it can be derived that*

$$\mathrm{Prob}\left(h_{k+i,k+i}=b_{i}\right)=\frac{b_{i}^{-(n+1-k-i)}}{\zeta (n+1-k-i)}.$$

**Proof.**  Since only one diagonal entry $a_{k+i}$ has been determined, the summation is for the remaining variables. By Lemma 1, we have

$$\begin{array}{cc} \; & Prob(h_{k+i,k+i}=b_{i})\\ = & \lim_{M\to +\infty }\frac{\left(\sum_{a_{1}\cdots a_{n-k-1}\leq \frac{M}{b_{i}}}a_{1}^{k}\cdots a_{i-1}^{k+i-2}a_{i}^{k+i}\cdots a_{n-k-1}^{n-1}\right)b_{i}^{k+i-1}}{|H_{n,k}^{\leq }\left(M\right)|}\\ = & \lim_{M\to +\infty }\frac{S\left(\frac{M}{b_{i}},k,\cdots ,k+i-2,k+i,\cdots ,n-1\right)b_{i}^{k+i-1}}{|H_{n,k}^{\leq }\left(M\right)|}\\ = & \lim_{M\to +\infty }\frac{\left(\frac{\prod_{j=2,\;j\neq n-k-i+1}^{n-k}\zeta \left(j\right)}{n}{\left(\frac{M}{b_{i}}\right)}^{n}+O\left({\left(\frac{M}{b_{i}}\right)}^{n-1}\log\frac{M}{b_{i}}\right)\right)b_{i}^{k+i-1}}{\frac{\prod_{j=2}^{n-k}\zeta \left(j\right)}{n}M^{n}+O\left(M^{n-1}\log M\right)}\\ = & \frac{b_{i}^{-(n+1-k-i)}}{\zeta (n+1-k-i)}. \end{array}$$

The proof is concluded.    □

**Remark** **2.** *Combining the above two theorems, we know that for sufficiently large $M\in Z^{+}$, the joint distribution of $h_{k+1,k+1},\cdots ,h_{n-1,n-1}$ is exactly the product of the distribution of each variable, which implies that these values can be viewed as independent variables.*

Since the distributions of $h_{k+1,k+1},\cdots ,h_{n-1,n-1}$ have been computed, the rejection sampling method can be utilized to generate them sequentially.

From Theorem 6, consider the distribution of $h_{k+i,k+i}$, if D(s) is defined as

(3) $$D\left(s\right):Prob\left(X=x\right)=\frac{1}{\zeta (s)}x^{-s}\;\left(x=1,2,\cdots \right),$$

then the distribution of $h_{k+i,k+i}$ is actually D(n+1−k−i).

To generate random variates following the distribution D(s), we employ the rejection sampling method. Since the target distribution D(s) has the density function f(x) as in Equation (3), we choose the proposal distribution $D^{\prime }$ over $Z^{+}$ with density

$$g\left(x\right)=\frac{1}{x(x+1)},\;\left(x=1,2,\cdots \right).$$

It could be verified that sampling from $D^{\prime }$ can be achieved by the simple transformation x=⌊1/v⌋, with v∼U[0,1]. Indeed, for any $x\in Z^{+}$, we have

$$\begin{array}{c} Prob\left(\left\lfloor 1/v\right\rfloor =x\right)=Prob\left(\frac{1}{x+1}<v\leq \frac{1}{x}\right)=\frac{1}{x}-\frac{1}{x+1}=g\left(x\right). \end{array}$$

Moreover, the maximum of $\frac{f(x)}{g(x)}=\frac{x^{-s}/\zeta \left(s\right)}{1/(x(x+1))}=\frac{x^{1-s}\left(x+1\right)}{\zeta (s)}$ is 2/ζ(s) attained at x=1, which implies the constant *C* can be set to 2/ζ(s). Thus the procedure of generating $h_{k+i,k+i}$ can be designed as follows.

1. Choose v∼U[0,1] and compute x=⌊1/v⌋;
2. Set s=n+1−k−i, compute $f\left(x\right)=x^{-s}/\zeta \left(s\right)$ and g(x)=1/x(x+1);
3. Choose v∼U[0,1]; when u>(ζ(s)f(x))/(2g(x)), reject and repeat from step 1; otherwise, accept and set $h_{k+i,k+i}=x$.

#### 5.2.2. Generating the Last Diagonal Entry $h_{nn}$

The case of generating the last diagonal entry $h_{nn}$ is different. Let *d* be the product of all diagonal entries except the last. Using the technique in [12], we can similarly obtain Theorem 7.

**Theorem** **7.** *For rank n≥4 and sufficiently large determinant upper bound $M\in Z^{+}$, given an (n,k)-random integer lattice with its HNF is $H=(h_{ij})$ and $d≜\prod_{i=1}^{n-1}h_{ii}$, we have*

$$\mathrm{Prob}(d\geq 1/{\left(\log M\right)}^{2})\leq 1/\log M.$$

**Sketch of the Proof.**  The proof consists of two main parts: deriving the mathematical expectation of *d* using the HNF counting lemma, and then applying Markov’s inequality to establish the probability upper bound.

For the first part, the expected value of the product of the first n−1 diagonal elements is formulated as

$$E\left(d\right)=\sum_{\leq M}t\cdot Prob\left(d=t\right)=\frac{\sum_{t\leq M}t\cdot \left|H_{n-1,k}\left(t\right)\right|\cdot \sum_{a_{n}\leq M/t}a_{n}^{n-1}}{|H_{n,k}^{\leq }\left(M\right)|},$$

where $|H_{n-1,k}\left(t\right)|$ represents the number of (n−1)-dimensional HNFs with determinant *t*, and $|H_{n,k}^{\leq }\left(M\right)|$ is the total number of (n,k)-simple HNFs with determinants upper bounded by *M*.

Expanding the numerator by using the HNF counting formula yields a multi-variable summation. This allows us to map both the numerator and the denominator directly to the asymptotic sums defined in Lemma 1 as

$$E\left(d\right)=\frac{S(M,k+1,k+2,\ldots ,n-1,n-1)}{S(M,k,k+1,\ldots ,n-2,n-1)}$$

According to the asymptotic formulas provided in Lemma 1, we have

$$\begin{array}{cc} \; & S\left(M,k+1,k+2,\ldots ,n-1,n-1\right)=\frac{\prod_{j=2}^{n-k-1}\zeta \left(j\right)}{n}M^{n}\log M+O\left(M^{n}\right),\\ \; & S\left(M,k,k+1,\ldots ,n-2,n-1\right)=\frac{\prod_{j=2}^{n-k}\zeta \left(j\right)}{n}M^{n}+O\left(M^{n-1}\log M\right). \end{array}$$

Dividing the above expressions yields

$$\begin{array}{cc} E(d) & =\frac{\frac{\prod_{j=2}^{n-k-1}\zeta \left(j\right)}{n}M^{n}\log M+O\left(M^{n}\right)}{\frac{\prod_{j=2}^{n-k}\zeta \left(j\right)}{n}M^{n}+O\left(M^{n-1}\log M\right)}\\ \; & =\frac{\log M+O(1)}{\zeta \left(n-k\right)+O\left(\frac{\log M}{M}\right)}\\ \; & =\frac{1}{\zeta (n-k)}\log M+O\left(1\right). \end{array}$$

This completes the first part of the proof.

For the second part, since $d=\prod_{i=1}^{n-1}h_{ii}$ is a product of positive diagonal entries, it is strictly a non-negative random variable. Markov’s inequality can be applied here, which states that for any non-negative random variable *X* and a constant a>0, it holds that

$$Prob\left(X\geq a\right)\leq \frac{E(X)}{a}.$$

By setting the random variable *d* and assigning the threshold parameter $a={\left(\log M\right)}^{2}$, we obtain the following tail probability bound as

$$Prob\left(d\geq {\left(\log M\right)}^{2}\right)\leq \frac{E(d)}{{\left(\log M\right)}^{2}}=\frac{\frac{1}{\zeta (n-k)}\log M+O\left(1\right)}{{\left(\log M\right)}^{2}}\leq \frac{1}{\log M},$$

which completes the second part of the proof.    □

Theorem 7 shows that $\prod_{i=1}^{n-1}h_{ii}$ is typically much smaller than *M*, which implies that ⌊M/d⌋ is still sufficiently large. Since the value range for $h_{nn}$ is large, we can use the inverse sampling method to obtain $h_{nn}$ by providing a reasonable characterization of the distribution of $h_{nn}$. Consequently, for an (n,k)-simple random integer lattice basis $H=(h_{ij})$, the distribution of $h_{nn}$ can be represented as

(4) $$\begin{array}{cc} \tilde{D}\left(n,M_{0}\right): & Prob(X=x)\\ = & \frac{1}{\sum_{t=1}^{M_{0}}t^{n-1}}x^{n-1}\;\left(x=1,2,\cdots ,M_{0}\right), \end{array}$$

which is actually a truncated discrete power-law distribution. Thus the distribution of $h_{nn}$ in an (n,k)-simple random integer lattice is actually $\tilde{D}\left(n,\left\lfloor M/d\right\rfloor \right)$ where $d=\prod_{i=1}^{n}h_{ii}$. After computing its cumulative distribution function F(x), we know that the following is a reasonable estimation of F(x) by considering the main terms as

$$F\left(x\right)≜Prob\left(X\leq x\right)\approx \frac{x^{n}/n}{M_{0}^{n}/n}={\left(\frac{x}{M_{0}}\right)}^{n}.$$

Since the function ${\left(\frac{x}{\left\lfloor M/d\right\rfloor }\right)}^{n}$ can be easily inverted, we can design the process of generating $h_{nn}$ as follows by the inverse sampling method.

1. Choose y∼U[0,1];
2. Compute *x* s.t. ${\left(\frac{x}{\left\lfloor M/d\right\rfloor }\right)}^{n}=y$, and set $h_{nn}=\left\lfloor x\right\rceil$.

Since the first *k* diagonal entries of (n,k)-simple HNF are 1, and the generation of the off-diagonal entries is easy, we can now propose the main generation algorithm for the (n,k)-simple random integer lattice.

### 5.3. Detailed Algorithm Description

After the discussion in Section 5.2, the detailed generation algorithm of (n,k)-simple random integer lattice is listed as Algorithm 1.
**Algorithm 1** Construction of an (n,k)-simple random integer lattice**Require:** lattice rank *n*, lattice determinant bound *M*, and k∈[1,n−1]**Ensure:** An (n,k)-simple random integer lattice L with det(L)≤M
    **Phase 1: Diagonal entries $h_{11},\ldots ,h_{n-1,n-1}$**    $h_{11}=\cdots =h_{kk}=1$    d←1    **for** i=1 **to** n−k−1 **do**        **repeat**            Sample v∼U[0,1] and set x←⌊1/v⌋            Compute acceptance probability $r\leftarrow x^{1-s}\left(x+1\right)$ with s=n+1−k−i            Draw u∼U[0,1]        **until** u≤r        d←d·x        $h_{k+i,\;k+i}\leftarrow x$    **end for**    **Phase 2: Final diagonal $h_{nn}$**    Sample y∼U[0,1]    $h_{nn}\leftarrow \left\lfloor \left\lfloor M/d\right\rfloor \cdot y^{1/n}\right\rceil$    **Phase 3: Off-diagonal entries**    **for** 
j=1 **to** 
*n* 
**do**        **for** i=1 **to** j−1 **do**            $h_{ij}∼U\left\{0,1,\ldots ,h_{jj}-1\right\}$        **end for**        **for** i=j+1 **to** *n* **do**            $h_{ij}\leftarrow 0$        **end for**    **end for**    **Phase 4: Output**    Construct $H=(h_{ij})$, return L(H)

### 5.4. Running Time Analysis of Algorithm 1

When analyzing the time complexity, notice that the time spent in Phase 2 and Phase 3 is obviously O(1) and $O(n^{2})$, respectively. Thus, the remaining task is to assess the repetition times in the repeat loop of Phase 1. In fact, the following theorem states the result.

**Theorem** **8.** *For lattice rank n and the parameter k∈[1,n−1], in each repeat loop of Algorithm 1, the expected number of iterations is bounded by 2.*

**Proof.**  Since within the repeat loop, the rejection sampling method is used, for s=n+1−k−i, we obtain that

$$\begin{array}{cc} \; & E(\mathrm{number}\;\mathrm{of}\;\mathrm{iterations})\\ = & 1/E(Prob(accept))\\ = & 1/\sum_{x\geq 1}g\left(x\right)\cdot \frac{f(x)}{Cg(x)}\\ = & C\cdot \sum_{x\geq 1}f\left(x\right)\\ = & C=\frac{2}{\zeta (s)}\leq 2, \end{array}$$

which completes the proof. □

By Theorem 8, since the expected number of iterations does not exceed 2, the expected total number of iterations within Phase 1 is bounded by 2(n−k−1), which is O(n). Thus, we have the following assessment of the time complexity of this algorithm.

**Theorem** **9.** *For lattice rank n and the parameter k∈[1,n−1], Algorithm 1 runs in $O(n^{2})$ expected time on average.*

## 6. Conclusions

In this work, the special (n,k)-simple HNF is first discussed; then the corresponding (n,k)-simple random integer lattice is defined and studied. Moreover, the probability result of the diagonal entries is utilized to construct the generation algorithm of (n,k)-simple random integer lattices. Finally, it is estimated that the generation algorithm takes $O(n^{2})$ time on average. However, an open problem to study in the future is the investigation of the distribution and density of random integer lattices whose HNF diagonal entries are restricted to specific prime powers, or whose off-diagonal entries satisfy certain linear congruence constraints. Exploring these structured variations would provide deeper insights into the random models required for Module-LWE and Ring-LWE variants in lattice-based cryptography.

## Figures and Tables

**Table 1:** The approximate density of (n,k)-simple integer lattices among all integer lattices (rounded to five decimal places).

*n*	k=1	$k=\frac{n}{4}$	$k=\frac{n}{2}$	$k=\frac{3n}{4}$	k=n−1
8	0.99609	0.99216	0.96145	0.76258	0.43574
16	0.99998	0.99976	0.99798	0.96074	0.43576
32	1.00000	0.99999	0.99999	0.99798	0.43576
64	1.00000	1.00000	1.00000	0.99998	0.43576
128	1.00000	1.00000	1.00000	1.00000	0.43576
256	1.00000	1.00000	1.00000	1.00000	0.43576

## Data Availability

No new data were created.
